# Influence of Different Solvents on the Mechanical Properties of Electrospun Scaffolds

**DOI:** 10.3390/ma18020355

**Published:** 2025-01-14

**Authors:** Dovydas Cicėnas, Andžela Šešok

**Affiliations:** Department of Biomechanical Engineering, Faculty of Mechanics, Vilnius Gediminas Technical University, Plytinės Str. 25, 10105 Vilnius, Lithuania; dovydas.cicenas@vilniustech.lt

**Keywords:** scaffold, nanofiber, electrospinning, polycaprolactone, solvent, mechanical properties

## Abstract

This article investigates the influence of different solvents on the mechanical properties of biocompatible and biodegradable polycaprolactone (PCL) scaffolds. During the research, using electrospinning technology, 27 samples of polycaprolactone nanofibers exposed to different solvents were produced. A tensile test was performed on the produced nanofiber samples, and the nanofiber mechanical properties, yield strength, elastic modulus, and elastic elongation were calculated, and load–displacement and stress–strain dependence diagrams were compared from the obtained results. The strongest nanofiber was singled out, and its mechanical properties were compared with those of biological tissues and its application in tissue engineering. The structure was determined using a scanning electron microscope, and the structures of nanofibers exposed to different solvents were compared. After calculating the influence of different solvents on the mechanical properties of the nanofibers, the strongest structure was identified, PCL and chloroform, which has an elastic modulus of 9.86 MPa and a yield strength of 1.11 ± 0.32 MPa. The type of solvent used in the production of the solution affects the homogeneity of the fibre and the shape of the filaments. In solvents with lower viscosity, the fibre filaments are more homogeneous and more evenly distributed.

## 1. Introduction

Proponents of new technologies are talking more and more about artificial biological tissues or organs. With advanced technology and medicine, tissue can be transplanted, and the process of tissue regeneration can be made more efficient. Tissue engineering and regenerative medicine are advancing rapidly to help people suffering from tissue loss and damage. Electrospinning is one of the most advanced methods of fabric fibre production, which uses an electric voltage to produce threads of polymer solutions or polymer melt fibres with a diameter of several hundred nanometres. During the construction of artificial tissues, cells are grown in vitro on synthetic or biological scaffolds and thus prepared for transplantation into the body [1].

During the last few decades, the electrospinning technique has been widely used for the production of polymer microcapsules and functional coatings on the surface of different materials. Compared to other conventional technologies, electrospinning overcomes major drawbacks such as agglomeration or particle size distribution. This technology is easily implemented and controlled, since the whole process is performed in one basic step, and the amount of toxic fumes emitted during electrospinning is small and effectively protected from entering the environment. Due to these advantages, electrospinning technology has recently been used for biomedical purposes in the development of scaffolds for tissue engineering [2]. Recent interest in electrospinning as a method to fabricate tissue engineering scaffolds is attributed to the relatively simple and inexpensive experimental setup, the wide variety of applicable materials, and the inherent nature of nanoscale fibres. One of the advantages of electrospinning nanofibrous scaffolds is that their surface can be modified by controlling the electrospinning parameters to obtain a topography that best suits the application. Another advantage is that nanofiber sheets or arrays can be formed into almost any shape (patches, mats, tubes, fibres, or multilayer arrays), depending on the desired implantation site. Further research and development of the electrospinning process and effective use of surface modification techniques will help create more biomimetic scaffolds [2,3].

Electrospinning is a suitable production technology to create scaffolds from different plastics [4]. Polycaprolactone (PCL) is widely used in various medical fields, such as drug delivery systems, and tissue engineering of ligaments, nerves, muscles, skin, and cardiovascular systems, as a result of its easy processing. In most of the applications, electrospun fibre scaffolds are used, in which the diameter and porosity of the fibres are not controlled. This method is suitable for the production of nano-scaffolds, as they meet the requirements of both porosity and strength, as tissues in the body are subjected to various and maximum loads. In addition, the properties of polycaprolactone meet most of the requirements for bone tissue—biocompatible, non-toxic, biodegradable, with good osteoconductive properties [5,6,7].

The electrospinning process can be applied in nanosensor production due to the possibility of creating extremely fine nanofibers with a high surface area to volume ratio, which is critical for the formation of sensitive surfaces. Nanofibers produced by this method can be used as a basis for various types of sensors, from biological and chemical analysis sensors to gas or moisture detectors. As a result of their highly porous structure, these fibres ensure the effective interaction of the materials with the analytes, which improves the sensitivity and accuracy of the sensors. By using functionalised polymers or nanoparticles integrated into the fibres formed during electrospinning, it is possible to create multipurpose sensors capable of recognising specific molecules or physical changes, such as temperature or light. Such structures are also lightweight, flexible, and easily integrated into more complex systems, making electrospinning technology indispensable for the development of a new generation of advanced sensors [8].

This technology is also very promising for the production of nanoreactors [9]. Electrospinning-produced nanostructures are ideal for the development of nanoreactors because their large surface area allows for the efficient distribution of catalysts and improves chemical reactions, e.g., in fuel cells or chemical sensors. Carbon-based nanoreactors exhibit improved electrolyte diffusion, more efficient charge transfer, and higher catalytic activity and selectivity compared to traditional particle-based catalysts. These properties open up new possibilities for the development of advanced energy storage, chemical, and environmental technologies. Furthermore, using advanced techniques such as coaxial electrospinning, it is possible to integrate different functional layers into a single fibre, which can be used as a multifunctional nanoreactor, e.g., by ensuring the controlled release of a chemical or protecting sensitive components from environmental influences.

The electrospinning technology meets the task set in this paper, taking into account the limitations of this technology in the production of nanofibers from polycaprolactone. The scaffolds of different structures can have different mechanical properties, depending on the amount and type of solvent used, porosity, the arrangement of layers, and the production parameters used, and it is not established which structure is the strongest, so it is necessary to study various structures to find a structure that has the strongest mechanical properties. In [10], it was found that the morphology of PCL is highly dependent on the solvents and operating parameters of the technology used. In this work, PCL microparticles and fibres were produced using electrohydrodynamic electrospray (EHD) and pressure spinning (PG) technologies. The authors used different solvents such as acetone, chloroform, dichloromethane, dimethylformamide, ethanol, methanol, tetrahydrofuran, and toluene. In addition, the authors used binary solutions using Chl together with Ace, MeOh, EtOh, and Dcm in three different ratios. In this work, the authors did not study the mechanical properties of the obtained nanofibers but focused mainly on the morphology. Solvent volatility affects the fibre diameter, while surface tension affects the morphology of bubbles on the nanowire, and vapour pressure affects the surface nanopores. The study found that gas pressure induced fibres, but at higher concentrations, thin fibres could become thicker, while reducing bubble and strand formation. Differences in solubility parameters also affected fibre morphology. The Chl:MeOh binary solvent system produced smaller, more uniform microparticles and improved fibre alignment compared to Chl alone. This work shows that PCL can be processed into various structures, such as particles and fibres, using electrospraying or pressure spinning, and the choice of solvent and operating conditions affect the nanofiber morphology [10].

The selection of a suitable solvent is a crucial step in the production of smooth and bead-free electrospun nanofibers [11]. PCL nanofibers can be obtained using different solvents such as chloroform, dimethylformamide, dichloromethane, and methanol or a combination of the mentioned solvents [12]. Chloroform is very often used for electrospinning. Some authors [13,14] emphasise that it is toxic and harmful. Recently, several research works have focused on less toxic and harmful solvents, such as acetic acid and acetone, for use in electrospinning [15,16]. These studies have shown an increased focus on less harmful solvents for electrospinning, but they have also shown that most of these solvents are not directly suitable for electrospinning. It is also not clear what influence the solvent used has on the mechanical properties of the manufactured scaffolds.

Most articles investigating the mechanical properties of scaffolds for tissue engineering are limited to not only compressive but also tensile tests [17]. This is because in the natural environment, the organs and tissues of an adult person experience not only compression, but also stretching and bending, so they are subjected to loads of human weight and movement, as well as various stresses from various directions with unstable and cyclic force [1].

The purpose of this paper is to investigate the influence of solvents used during electrospinning technology and their concentrations on the mechanical properties of the scaffolds obtained and to evaluate the suitability of the mechanical properties of the produced nanofibers in tissue engineering.

## 2. Materials and Methods

PCL with an average molecular weight of 80,000 was purchased from Sigma-Aldrich (St. Louis, MO, USA). Polycaprolactone is a biodegradable plastic obtained from the polymers of natural origin, which is degraded by living organisms, usually bacteria. PCL (polycaprolactone) is used in medicine and tissue engineering, such as bioprinting. PCL has a melting point of 65 °C and has good mechanical properties, making it useful for bone and cartilage tissue regeneration.

Chloroform, acetone, and acetic acid (Sigma–Aldrich) were used as solvents. Chloroform was chosen due to the good solubility of PCL in this solvent. However, it is toxic. Therefore, two other solvents (acetone and acetic acid) were chosen for comparison, which are classified as “green” [15]. Electrospinning solutions are prepared by dissolving an appropriate amount of PCL granules in each of the solvents used in this study (chloroform, acetone, and acetic acid) at 40 °C with stirring for 1 h to completely dissolve the PCL granules. Three concentrations of PCL (10 wt%, 12 wt%, and 15 wt%) are prepared for each solvent. Thus, a total of nine solutions are prepared. To perform mechanical tests, 3 nanofiber samples (27 in total) are produced for each concentration of solution. Before electrospinning, each solution was cooled to room temperature (25 °C). Because chloroform is harmful to health, the production of solutions is carried out in a fume hood in the laboratory. The prepared solutions were kept under the same conditions.

**Figure 1:**
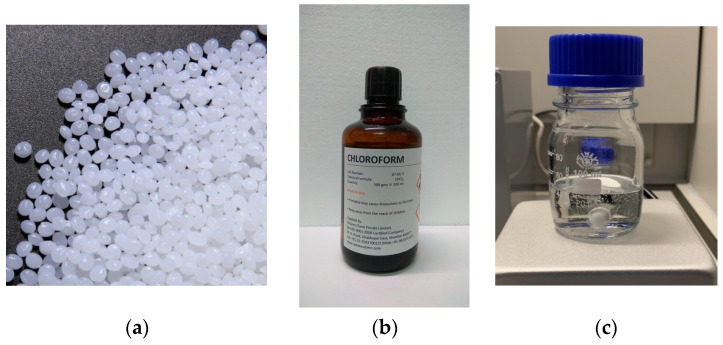
Materials and production of solution: (**a**) PCL granules; (**b**) solvent (chloroform); (**c**) the prepared solution.

The mixing vessel is placed on a stirrer with a heating tile that catalyses the dissolution process of PCL granules in the solvent. The mixer is turned on, and the magnet capsule inserted into the solution performs the mixing process. The materials and solution production process are shown in Figure 1.

Spinbox of Electrospinning Equipment (Spinbox System, Bionica S. L. Paterna, Valencia, Spain) (Vilnius Gediminas Technical University) is used to produce scaffolds. A critical ‘spinning’ concentration was used, which is defined as the lowest concentration below which electrospinning beads will form, rather than electrospun fibres. However, if the solution were too concentrated, the high viscosity would also inhibit the flow to the needle tip and slow the electrospinning process. Thus, in this case, the solutions used in this study were prepared taking into account the ‘critical concentration’ at which the electrospinning voltage and the distance to the collector were selected in parallel. The parameters of the electrospinning equipment are set to be fixed: voltage at 20 kV, distance between collector and emitter is 20 cm, flow rate of 3.5 mL/h. The environmental conditions during the electrospinning process are an ambient temperature of 18–25 °C and a relative humidity of 40–50%.

**Figure 2:**
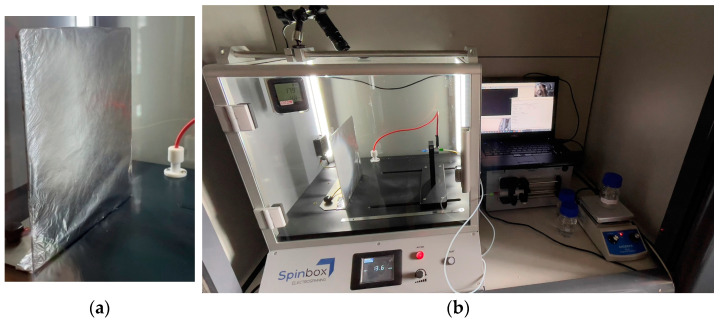
The electrospinning device: (**a**) collector with aluminium foil layer; (**b**) the Spinbox electrospinning device.

A 20 mL medical syringe with PCL solution is inserted into the automatic infusion pump. The collector is covered with a layer of aluminium foil (Figure 2a). It is very important that the aluminium foil on the collector is smooth because the nanofiber integrity of the produced nanofibers depends on it. The spray needle is placed at a distance of 20 cm from the collector. A fixed voltage of 20 kV is established. The general view of the ready-to-use Spinbox device is shown in Figure 2b.

The nanofibers formed by the Spinbox electrospinning device are prepared for the study of mechanical properties. The length, width, and height of the obtained fibre samples obtained are measured with a calliper with an accuracy of 0.05 mm. The dimensional error of the formed nanofibers should not exceed ±0.2.

**Figure 3:**
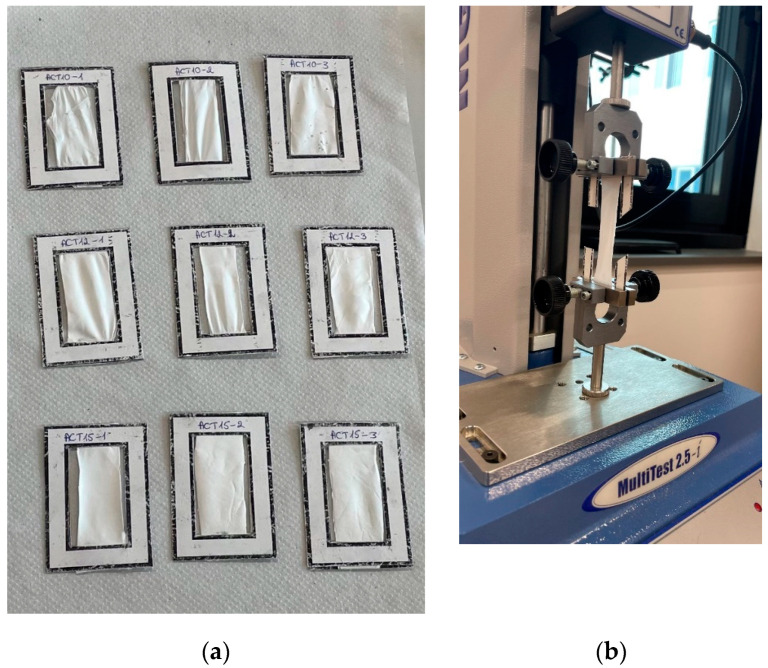
Mechanical properties test: (**a**) PCL scaffolds exposed to chloroform (CL) prior to tensile testing; (**b**) sample in the Mecmesin MultiTest 2.5-i mechanical properties testing machine.

Nanofibers are very thin and delicate, making them difficult to prepare for testing. To avoid damage to the sample, a paper frame is used (Figure 3a). The tensile rate of the specimen is determined according to the recommendations of BS EN ISO 9073 “Nonwovens. Test methods” [18], taking into account the length and width of the specimen. In this study, the dimensions of the nanofiber samples are 43 mm × 24 mm. According to the standard BS EN ISO 9073-2, a speed of 15 mm/min and a breaking load of 24 N are recommended to stretch this sample. The tensile test is performed using the ‘Mecmesin MultiTest 2.5-i’ mechanical properties test machine, Mecmesin Limited, Slinfold, UK (Figure 3b). The sample is placed between two standard clamps. After that, the paper frame of the sample is cut, and the test is started.

Before the tensile test, all samples are assigned an identification number based on solvent (CL, chloroform; ACE, acetic acid; ACT, acetone), solvent concentration (10%, 12%, and 15%) and specimen number (1, 2, and 3).

Data were presented as mean ± standard deviation. One-way ANOVA was used to compare the mean values of each sample group. Values of *p* < 0.05 indicated a significant difference. To assess the reliability of the results, a confidence interval was calculated. The following values are required to calculate it: the chosen level of confidence is 95%, which means that the significance level is *p* < 0.05; the number of degrees of freedom is calculated by subtracting one from the data sample, and in this case 9 samples of each structure were tested; therefore, the number of degrees of freedom is equal to 8; the critical value, the value of which is taken from the statistical manual, taking into account the significance level and the number of degrees of freedom. This value is 2.306.

The morphology of the nanofibers produced, and the diameters of the nanowires are studied with a Hitachi S-3400N scanning electron microscope (SEM), Hitachi, Tokyo, Japan. Morphological analysis of nanofibers is performed using an accelerating voltage of 10 kV and an image magnification of 25 to 1000 times. After surface scanning of the nanofibers, nanowire diameter analysis is performed by randomly counting 50 filaments per sample using ImageJ 1.54d software. The average diameter and standard deviation are calculated from the obtained results.

## 3. Results

The parameters of the electrospun nanofiber scaffolds were influenced by the temperature and concentration of the electrospun environment and the solution. It was observed that, at a high solution temperature and a higher solution concentration, the first layers of nanofibers tend to stick to the base of the printing plane (collector). The concentration of the solution determined the area and integrity of the samples.

**Figure 4:**
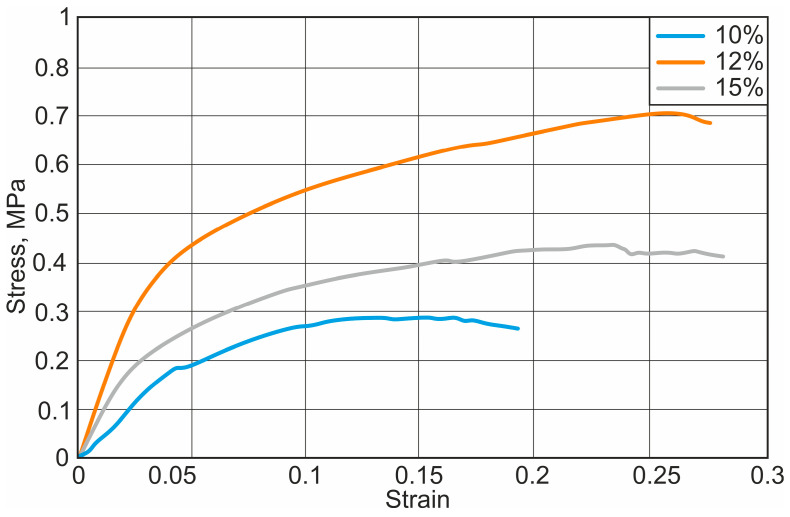
Stress–strain diagram for PCL and chloroform nanofibers: 10%, 12%, and 15%—PCL concentrations.

**Figure 5:**
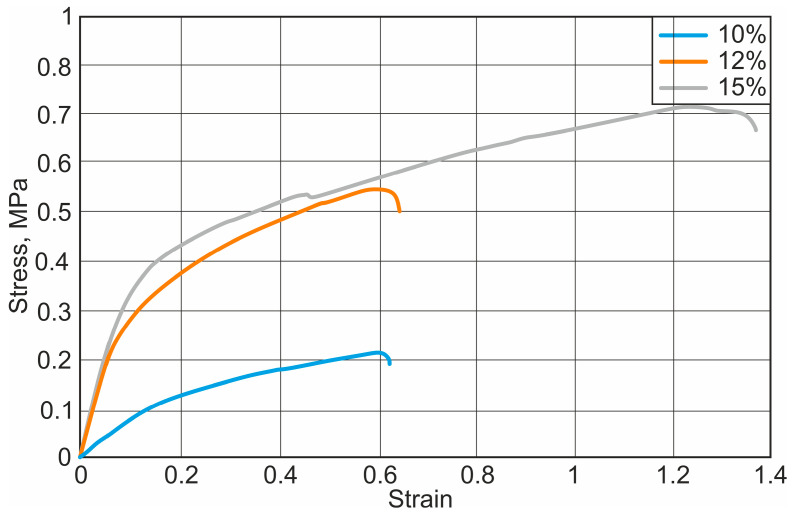
Stress–strain diagram for PCL and acetone nanofibers: 10%, 12%, and 15%—PCL concentrations.

**Figure 6:**
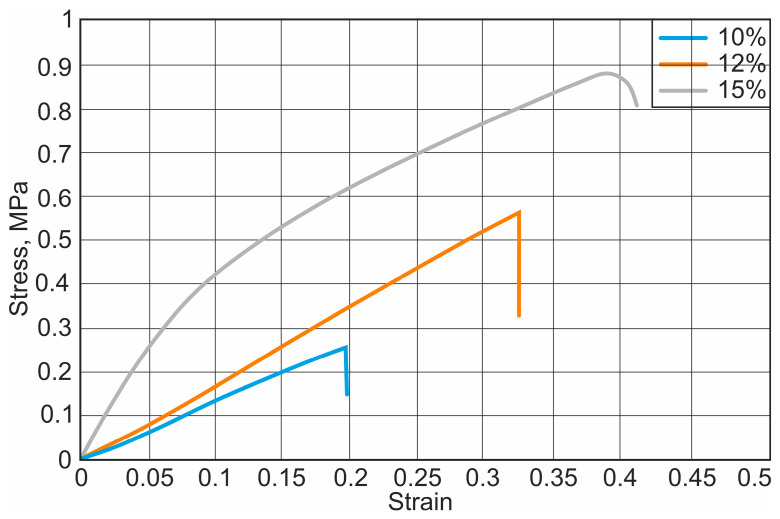
Stress–strain diagram for PCL and acetic acid nanofibers: 10%, 12%, and 15%—PCL concentrations.

After the uniaxial tensile test with the Macmesin MultiTest 2.5-i mechanical parameters testing machine, the initial curves were obtained, showing the relationship between the load applied to the specimen and the displacement of the specimen. The stress–strain curves of different nanofibers are presented in Figure 4, Figure 5 and Figure 6.

It has been observed that the mechanical properties vary depending on the concentration of the solution. Fibres with higher concentrations had higher strength and ductility. The results of the study show that the mechanical strength of the nanofibrous scaffolds increases with increasing solvent concentration. The yield strength increases from 0.52 MPa to 1.11 MPa and the elastic modulus from 1.3 MPa to 9.86 MPa, respectively. Depending on the type and concentration of solvent, the plasticity of the fibre increases on average by about 69.8%. The calculated results of the elastic modulus, yield strength, and elastic elongation are presented in Table 1.

After calculating the parameters of the mechanical properties of different nanofibers, the PCL and chloroform sample stood out with the highest elastic modulus, the average elastic modulus of which was 6.53 MPa. The weakest nanofiber was the PCL and acetic acid sample, which had the lowest yield strength. The PCL and chloroform samples have the highest modulus of elasticity. The results are statistically significant at a significance level of *p* < 0.05.

**Figure 7:**
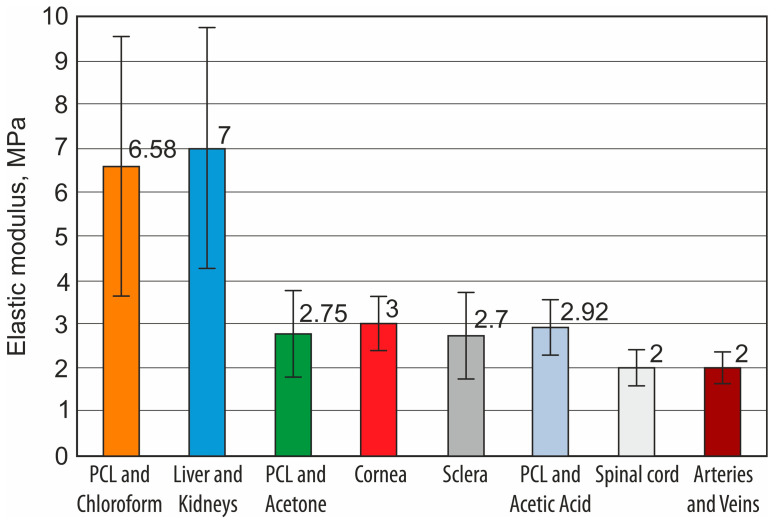
Comparison of the elastic modulus of electrospun polycaprolactone nanofibrous scaffolds.

The suitability of artificial tissue scaffolds in tissue engineering is evaluated by their mechanical properties. The main properties are the modulus of elasticity and yield strength. Scaffolds are suitable only if the mechanical properties are close to the mechanical properties of the natural tissues. Figure 7 shows a comparison of the elastic modulus of the printed polycaprolactone nanofibers with the values of the tissue elastic modulus reported in the literature.

When comparing the average values of the modulus of elasticity of biological tissues with the modulus of elasticity of nanofiber scaffolds obtained during the study, it was observed that the modulus of elasticity of PCL and chloroform corresponds to the modulus of elasticity of the biological tissues of liver and kidney walls by 98.6%. The value of the elastic modulus of this nanofiber is the highest. The PCL and acetone nanofiber scaffold had the second-highest modulus value, with an average modulus value equal to that of the cornea, and it can also be used in tissue engineering as replacement tissue for the sclera, spinal cord, arteries, and veins. The third nanofiber structure obtained in the study from PCL and acetic acid had an elastic modulus equal to the elastic moduli of the arteries, veins, spinal cord, and grey matter tissues. When the process parameters and increasing of the thickness of the nanofibers increased during electrospinning, structures that can be used in tissue engineering to replace human skin can be easily achieved.

The limits of the elastic modulus of the skin are 21–39 MPa, so it would not be difficult to reach such values. After evaluating the results of the study, it can be stated that the values of the elastic modulus are sufficient to produce tissues of these organs, which can be produced from nanofiber frameworks in tissue engineering.

**Figure 8:**
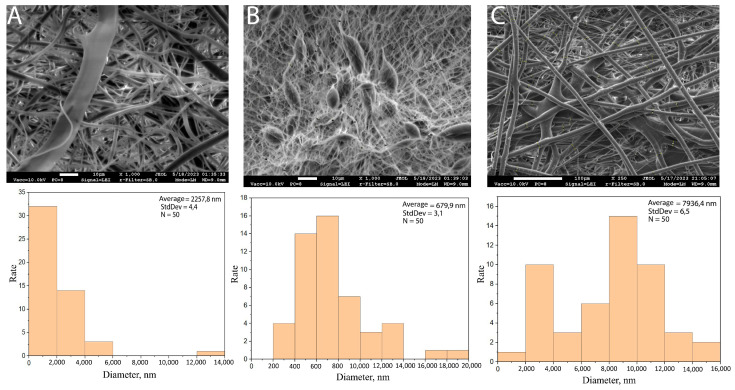
Comparison of the elastic modulus of electrospun polycaprolactone nanofibrous scaffolds: (**A**) PCL and acetone; (**B**) PCL and acetic acid; (**C**) PCL and chloroform.

The type of solvent influences the structure of the nanofibers (Figure 8).

In the SEM images, it can be observed that the morphology of the polycaprolactone nanofibers in which chloroform was used as a solvent was granule-free and homogeneous. Fibres of larger diameter were obtained by electrospinning a PCL solution with a chloroform solvent. The diameters of these fibres were also distributed over a wide range of values. This nanofiber also differs from others in its morphology: The filaments are regular in shape, arranged in orderly layers, and are formed because of the lower viscosity of chloroform. Compared with PCL nanofibers exposed to acetic acid, irregularly shaped fibre strands with PCL polymer clusters are observed in the latter.

The high viscosity of the acetic acid caused the polymer to clog at the end of the needle, a common problem encountered in electrolysis. This made the electrospinning process quite difficult and resulted in uneven fibres of irregular shape.

The evaporation rate of solvents is one of the most important factors in determining the morphology of nanofibers during electrospinning. Fast-evaporating solvents, such as acetone, can promote faster fibre solidification, but at the same time, cause surface irregularities or pore formation because the components in the solution do not have enough time to evenly distribute. Slower-evaporating solvents, such as dimethylformamide (DMF), allow for the formation of a smoother and more homogeneous fibre structure due to the gradual solvent release and lower surface tension. The use of mixtures using solvents with different evaporation rates can also help to control morphological properties, such as porosity or fibre diameter. These properties are crucial in applications where a precise level of surface control is required, such as in filtration membranes or drug delivery systems. Optimal control of the evaporation rate helps avoid fibre defects such as droplet formation, fibre filament adhesion, or the formation of fibres with improper structure.

## 4. Discussion

In contrast to the mechanical results of the nanofibers obtained in the present study, several other studies of electrospun PCL scaffolds have shown a much higher ultimate strength range of 500 to 1000 MPa. Studies of randomly orientated electrospun PCL showed even lower ultimate tensile strength values of 1–2.38 MPa and elastic modulus values of 8.5–31 MPa [19,20,21,22]. Although the current study does not exceed the mentioned values, there were other studies in the reviewed literature that generally showed similar (lower) values of mechanical parameters (1–25 MPa) for PCL nanofibers after spinning [23]. The quantitative analysis of the cross-sectional area and the evaluation of the mechanical properties may have influenced the results of the study. Differences in the parameters of the mechanical properties of nanofiber scaffolds can be explained by several reasons.

1. Mechanical anisotropy is the main factor affecting the mechanical properties of nanofibers. When the nanofibers are randomly distributed, the fibre strength is lower than that of orientated nanofibers. Moreover, when the solvent concentration is higher, the resulting nanofibers exhibit better alignment orientation. The orientation of the nanowires increases the strength of the nanofibrous scaffolds;
2. Studies show that a decrease in the fibre diameter of about 700 nm results in a change in the modulus of elasticity. This change becomes even more pronounced when the nanofiber diameter is less than 700 nm. The studies conducted showed that as the diameter of the fibre decreases to a critical value, the mechanical properties increase exponentially. When the diameter of the nanofiber is entangled between the molecules, the nanofiber structure will have fewer defects and a more uniform structure;
3. The mechanical parameters of nanofibers depend on many factors, such as the chemical structure of the polymer, molecular orientation, etc. PCL is an amorphous polymer. The random or ordered arrangement of the amorphous phase determines the physical and mechanical properties of the nanofibers. In the electrospinning process, the polymer molecules are pulled apart under the action of electromagnetic forces, creating the orientation of the polymer molecule. This increases the strength of the nanofiber scaffolds.

In conclusion, it can be said that the improvement in the mechanical parameters of the nanofibers is due to the arrangement of nanowires in the fibres, the diameter of the fibre, and the molecular orientation in the amorphous phase of the polymer. Recently, researchers have formed vascular grafts from nanofibers derived from mixed fibres of fibrinogen and PCL. The grafts exhibited robust mechanical properties and a biomimetic extracellular matrix (ECM) arrangement with a rich expression of elastic fibre proteins. These results show promise for the future use of composite nanofibers [24,25]. Furthermore, future studies may focus on unravelling the molecular mechanism that causes these mechanical properties. From a materials science perspective, the effect of nanofiber geometry on fibre properties should be an interesting topic. Polymeric nanofibers can be processed by various methods, such as templated synthesis, phase separation, self-assembly, and electrospinning. A major advantage of electrospinning is that long and continuous nanofibers can be produced in a cost-effective manner, making them suitable for industrial processing. However, the drawback that it shares with other nanotechnologies is low productivity. To be commercially viable, it is necessary to increase the rate of nanofiber production. To do this, a multispinning setup is used to increase product yield, such as the use of eight needles per syringe pump and a feed rate of 24 mL / min for the production of PCL nanofibers [21].

## 5. Conclusions

1. Concentration affects solution viscosity and surface tension, both of which can affect the electrospinning process. However, if the solution is too concentrated, the high viscosity will also inhibit the flow of the liquid to the needle and, therefore, slow down the electrospinning process.
2. The type of solvent and its concentration in the polycaprolactone (PCL) solution used in electrospinning technology to produce the nanofiber affect the mechanical properties of the scaffolds.
3. After calculating the influence of different solvents on the mechanical properties of the nanofibers, the strongest structure was selected, PCL and chloroform, which has an elastic modulus of 9.86 MPa and a yield strength of 1.11 ± 0.32 MPa.
4. The nanofiber of the strongest sample is close to the mechanical properties of the liver and kidney walls by up to 98.6%. The mechanical properties of the other two different nanofibers correspond to the values of the mechanical parameters of the soft tissues.
5. The mechanical properties of PCL nanofibers exposed to acetone and acetic acid are very close to the mechanical properties of blood vessels, brain, cornea, and sclera, and some concentrations of nanofibers exceed the values of the mechanical parameters of these biological tissues.
6. After the analysis of the morphological structure of the nanofibers was performed with an SEM, it was observed that the type of solvent used in the production of the solution affects the homogeneity of the fibre and the shape of the filaments. In solvents with lower viscosity, the fibre filaments are more homogeneous and more evenly distributed.

## Figures and Tables

**Table 1:** Calculated mechanical parameter values for different nanofibers.

Sample	Elastic Modulus, MPa	Yield Strength, MPa	Elastic Elongation, %
PCL and chloroform	6.53 ± 3.33	1.11 ± 0.32	47.6 ± 20
PCL and acetone	2.75 ± 1.45	0.78 ± 0.26	113 ± 33
PCL and acetic acid	2.92 ± 1.15	0.52 ± 0.32	48.9 ± 16

## Data Availability

The original contributions presented in this study are included in the article. Further inquiries can be directed to the corresponding author.
